# Supplementary material for: The role of chromatin dynamics under global warming response in the symbiotic coral model Aiptasia
Source: Commun Biol. 2019 Aug 2;2:282. doi: 10.1038/s42003-019-0543-y (PMC6677750; doi:10.1038/s42003-019-0543-y)
Supplement: Supplementary file 1 — Supplementary Information [file 42003_2019_543_MOESM1_ESM.pdf]

Supplementary Figures:

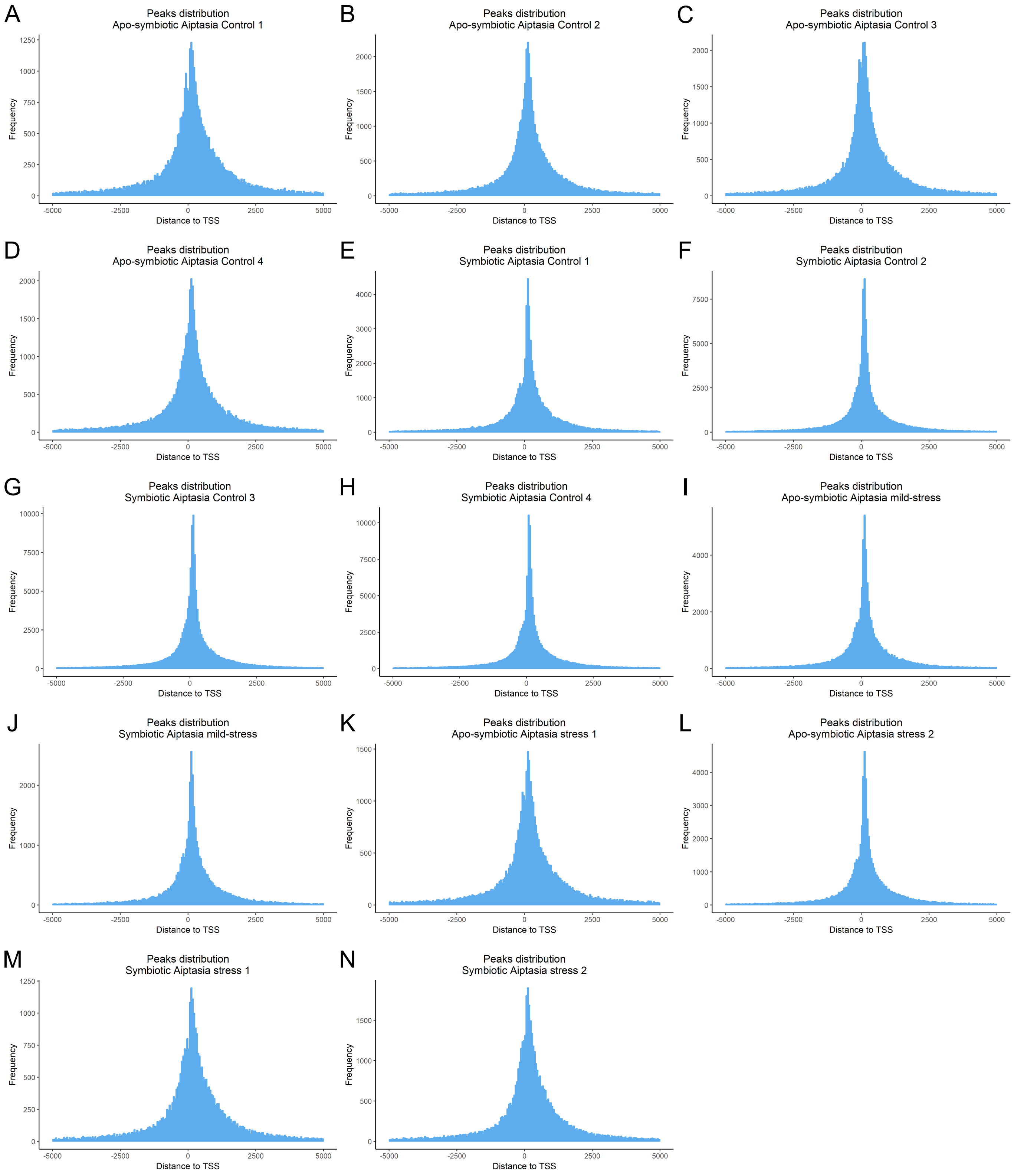

Supplementary Figure S 1: Histograms of distance from the nearest transcriptional start site (TSS) for all ATAC-seq peaks within 5 kb of the nearest TSS that were identified in all group tested.

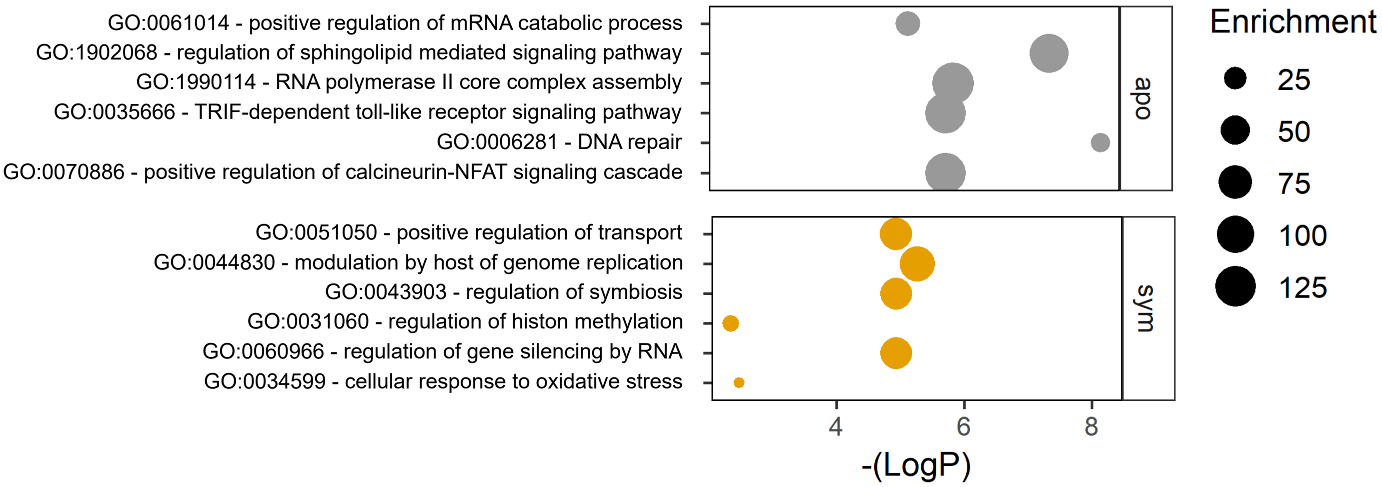

Supplementary Figure 2: GO enrichment analysis of unique accessible site of symbiotic and apo-symbiotic aiptasia at basal conditions (day 0; 24C°). In grey apo-symbiotic aiptasia. In yellow symbiotic aiptasia. Dots size represent biological proses relative enrichment.

Supplementary Tables:

Supplementary Table 1: Temperature and sampling regimen					
**Day**	**Apo Aiptasia (Stress) C°**	**Apo Aiptasia (Control) C°**	**Symbiotic Aiptasia (Stress) C°**	**Symbiotic Aiptasia (Control) C°**	**Sampling**
Day 0	24	24	24	24	sampled
Day 1	24.5	24	24.5	24	---
Day 2	25	24	25	24	---
Day 3	25.5	24	25.5	24	---
Day 4	26	24	26	24	---
Day 5	26.5	24	26.5	24	---
Day 6	27	24	27	24	---
Day 7	27.5	24	27.5	24	---
Day 8	28	24	28	24	---
Day 9	28	24	28	24	sampled
Day 10	28.5	24	28.5	24	---
Day 11	29	24	29	24	---
Day 12	29.5	24	29.5	24	---
Day 13	30	24	30	24	---
Day 14	30.5	24	30.5	24	---
Day 15	31	24	31	24	---
Day 16	31.5	24	31.5	24	---
Day 17	32	24	32	24	---
Day 18	32.5	24	32.5	24	---
Day 29	33	24	33	24	---
Day 20	33.5	24	33.5	24	---
Day 21	34	24	34	24	sampled
Day 22	34	24	34	24	---
Day 23	34	24	34	24	---
Day 24	34	24	34	24	---
Day 25	34	24	34	24	---
Day 26	34	24	34	24	---
Day 27	34	24	34	24	---
Day 28	34	24	34	24	sampled

Supplementary Table 2 - Primers used to amplify ATAC-seq libraries	
Ad1_noMX:	AATGATACGGCGACCACCGAGATCTACACTCGTCGGCAGCGTCAGATGTG
Ad2.1_TAAGGCGA	CAAGCAGAAGACGGCATACGAGATTCGCCTTAGTCTCGTGGGCTCGGAGATGT
Ad2.2_CGTACTAG	CAAGCAGAAGACGGCATACGAGATCTAGTACGGTCTCGTGGGCTCGGAGATGT
Ad2.3_AGGCAGAA	CAAGCAGAAGACGGCATACGAGATTTCTGCCTGTCTCGTGGGCTCGGAGATGT
Ad2.4_TCCTGAGC	CAAGCAGAAGACGGCATACGAGATGCTCAGGAGTCTCGTGGGCTCGGAGATGT
Ad2.5_GGACTCCT	CAAGCAGAAGACGGCATACGAGATAGGAGTCCGTCTCGTGGGCTCGGAGATGT
Ad2.6_TAGGCATG	CAAGCAGAAGACGGCATACGAGATCATGCCTAGTCTCGTGGGCTCGGAGATGT
Ad2.7_CTCTCTAC	CAAGCAGAAGACGGCATACGAGATGTAGAGAGGTCTCGTGGGCTCGGAGATGT
Ad2.8_CAGAGAGG	CAAGCAGAAGACGGCATACGAGATCCTCTCTGGTCTCGTGGGCTCGGAGATGT
