# Supplementary material for: The role of chromatin dynamics under global warming response in the symbiotic coral model Aiptasia
Source: Commun Biol. 2019 Aug 2;2:282. doi: 10.1038/s42003-019-0543-y (PMC6677750; doi:10.1038/s42003-019-0543-y)
Supplement: Supplementary file 2 — Reporting Summary [file 42003_2019_543_MOESM2_ESM.pdf]

Corresponding author(s): Eviatar Weizman and Oren Levy

Last updated by author(s): Jun 25, 2019

Reporting Summary

Nature Research wishes to improve the reproducibility of the work that we publish. This form provides structure for consistency and transparency in reporting. For further information on Nature Research policies, see Authors & Referees and the Editorial Policy Checklist.

Statistics

For all statistical analyses, confirm that the following items are present in the figure legend, table legend, main text, or Methods section.

n/a	Confirmed	
☐	☒	The exact sample size (*n*) for each experimental group/condition, given as a discrete number and unit of measurement
☐	☒	A statement on whether measurements were taken from distinct samples or whether the same sample was measured repeatedly
☐	☒	The statistical test(s) used AND whether they are one- or two-sided<br>*Only common tests should be described solely by name; describe more complex techniques in the Methods section.*
☒	☐	A description of all covariates tested
☐	☒	A description of any assumptions or corrections, such as tests of normality and adjustment for multiple comparisons
☐	☒	A full description of the statistical parameters including central tendency (e.g. means) or other basic estimates (e.g. regression coefficient) AND variation (e.g. standard deviation) or associated estimates of uncertainty (e.g. confidence intervals)
☐	☒	For null hypothesis testing, the test statistic (e.g. *F*, *t*, *r*) with confidence intervals, effect sizes, degrees of freedom and *P* value noted<br>*Give P values as exact values whenever suitable.*
☒	☐	For Bayesian analysis, information on the choice of priors and Markov chain Monte Carlo settings
☐	☒	For hierarchical and complex designs, identification of the appropriate level for tests and full reporting of outcomes
☐	☒	Estimates of effect sizes (e.g. Cohen's *d*, Pearson's *r*), indicating how they were calculated

Our web collection on statistics for biologists contains articles on many of the points above.

Software and code

Policy information about availability of computer code

Data collection	Bowtie 2 version 2.3.2 by Ben Langmead (langmea@cs.jhu.edu, www.cs.jhu.edu/~langmea).<br>STAR version 2.6.0a by Alex Dobin, dobin@cshl.edu https://groups.google.com/d/forum/rna-star<br>MACS2 version 2.1.1 by Yong Zhang and Tao Liu from Xiaole Shirley Liu's Lab.
Data analysis	DESeq2 package version: 1.23.9 by Bioconductor.<br>HOMER Software for motif discovery and next-gen sequencing analysis by Chris Benner et al.<br>IGB - Nicol, J. W., Helt, G. A., Blanchard Jr, S. G., Raja, A., & Loraine, A. E. (2009). The Integrated Genome Browser: free software for distribution and exploration of genome-scale datasets. Bioinformatics, 25(20), 2730-2731.<br>metascape - Zhou et al., Nature Communications 2019, 10(1):1523 http://metascape.org

For manuscripts utilizing custom algorithms or software that are central to the research but not yet described in published literature, software must be made available to editors/reviewers. We strongly encourage code deposition in a community repository (e.g. GitHub). See the Nature Research guidelines for submitting code & software for further information.

Data

Policy information about availability of data

All manuscripts must include a data availability statement. This statement should provide the following information, where applicable:

- Accession codes, unique identifiers, or web links for publicly available datasets
- A list of figures that have associated raw data
- A description of any restrictions on data availability

The sequencing data reported in this study has been deposited to the Sequence Read Archive (SRA) BioProject, under accession: PRJNA518019.

Field-specific reporting

Please select the one below that is the best fit for your research. If you are not sure, read the appropriate sections before making your selection.

☒ Life sciences ☐ Behavioural & social sciences ☐ Ecological, evolutionary & environmental sciences

For a reference copy of the document with all sections, see nature.com/documents/nr-reporting-summary-flat.pdf

Life sciences study design

All studies must disclose on these points even when the disclosure is negative.

Sample size	We have sampled and sequenced 3 biological replicates from 3 Aiptasia individuals for ATAC-seq library preparations as accepted.<br>for RNA seq we pooled together 10 aiptasia individuals per sampling point and group. we have sequenced RNA libraries from 3 individual Aipatsia for pools validation.
Data exclusions	No data was exclude from analysis
Replication	For ATAC-seq we have used 3 biological replicats.<br>For RNA-seq we have used pools of 10 animals and compared them to 3 libraries from individual animals for validation
Randomization	animals were divided randomly between groups.
Blinding	no blinding was preformed in our work.

Reporting for specific materials, systems and methods

We require information from authors about some types of materials, experimental systems and methods used in many studies. Here, indicate whether each material, system or method listed is relevant to your study. If you are not sure if a list item applies to your research, read the appropriate section before selecting a response.

Materials & experimental systems

n/a	Involved in the study
☒	☐ Antibodies
☒	☐ Eukaryotic cell lines
☒	☐ Palaeontology
☐	☒ Animals and other organisms
☒	☐ Human research participants
☒	☐ Clinical data

Methods

n/a	Involved in the study
☒	☐ ChIP-seq
☒	☐ Flow cytometry
☒	☐ MRI-based neuroimaging

Animals and other organisms

Policy information about studies involving animals; ARRIVE guidelines recommended for reporting animal research

Laboratory animals	we have used Exaiptasia pallida sea anemone
Wild animals	NA
Field-collected samples	study did not involved samples collected from field
Ethics oversight	Working with cnidaria does not require ethics oversight

Note that full information on the approval of the study protocol must also be provided in the manuscript.
